# Can activated long-term memory maintain serial order information?

**DOI:** 10.3758/s13423-021-01902-3

**Published:** 2021-03-25

**Authors:** Benjamin Kowialiewski, Benoît Lemaire, Steve Majerus, Sophie Portrat

**Affiliations:** 1 Department of Psychology, University of Zurich, Binzmühlestrasse 14, 8050, Zürich, Switzerland; 2 University of Liège, Liège, Belgium; 3 Laboratoire de Psychologie et NeuroCognition (LPNC), Université Grenoble Alpes, Bâtiment Michel Dubois prev. BSHM, 1251 Avenue Centrale, 38400 Saint-Martin-d’Hères, France; 4 Fund for Scientific Research – F.R.S.-FNRS, Brussels, Belgium

**Keywords:** Working memory, Serial order, Computational modeling, Semantic knowledge

## Abstract

The maintenance of serial order information is a core component of working memory (WM). Many theoretical models assume the existence of specific serial order mechanisms. Those are considered to be independent from the linguistic system supporting maintenance of item information. This is based on studies showing that psycholinguistic factors strongly affect the ability to maintain item information, while leaving order recall relatively unaffected. Recent language-based accounts suggest, however, that the linguistic system could provide mechanisms that are sufficient for serial order maintenance. A strong version of these accounts postulates serial order maintenance as emerging from the pattern of activation occurring in the linguistic system. In the present study, we tested this assumption via a computational modeling approach by implementing a purely activation-based architecture. We tested this architecture against several experiments involving the manipulation of semantic relatedness, a psycholinguistic variable that has been shown to interact with serial order processing in a complex manner. We show that this activation-based architecture struggles to account for interactions between semantic knowledge and serial order processing. This study fails to support activated long-term memory as an exclusive mechanism supporting serial order maintenance.

## Introduction

The ability to maintain serial order information is a core component of verbal working memory (WM). Mechanisms involved in the maintenance of serial order (i.e., the sequential order of the to-be-remembered items) have been considered to be independent from those involved in the maintenance of item information (i.e., the linguistic content of the to-be-remembered items). This assumption is supported by different lines of research. Studies examining the impact of psycholinguistic factors, such as lexicality, on verbal WM performance typically observe effects on item recall, with minimal effects on serial order recall (Allen & Hulme, 2006; Hulme, 2003; Romani, Mcalpine, & Martin, 2008; Roodenrys, Hulme, Lethbridge, Hinton, & Nimmo, 2002; Saint-Aubin & Ouellette, 2005; Walker & Hulme, 1999). In addition, serial order-recall performance is more strongly affected by rhythmic and articulatory interfering tasks than is the maintenance of item information (Gorin, Kowialiewski, & Majerus, 2016; Henson, Hartley, Burgess, Hitch, & Flude, 2003). Neuropsychological studies have also reported the existence of double dissociations between serial order and item-recall performance in several brain-injured patients and populations affected by neurodevelopmental disorders (Brock & Jarrold, 2005; Majerus, Attout, Artielle, & Kaa, 2015; Martinez Perez, Poncelet, Salmon, & Majerus, 2015). Finally, the maintenance of item and serial order information is supported by different neural substrates, as reported by neurostimulation and neuroimaging studies (Attout, Fias, Salmon, & Majerus, 2014; Guidali, Pisoni, Bolognini, & Papagno, 2019; Kalm & Norris, 2014; Majerus et al., 2010; Papagno et al., 2017).

At the same time, other studies suggest that serial order recall can also interact with linguistic knowledge. Although lexical knowledge strongly enhances recall of item information, it also constrains phoneme migration errors within and between items (Jefferies, Frankish, & Lambon Ralph, 2006). Likewise, nonwords, even if more poorly recalled as compared to words at the item level, can show a relative advantage regarding serial order recall (Fallon, Mak, Tehan, & Daly, 2005; Kowialiewski & Majerus, 2018; Saint-Aubin & Poirier, 1999). Recently, Kalm and Norris (2014) showed that the serial order of nonwords could be decoded on the basis of neural patterns elicited within the dorsal language pathways supporting encoding and maintenance of verbal information. Similarly, Papagno et al. (2017) showed that serial order-recall performance decreases, as compared to item-recall performance, when the posterior part of the dorsal language pathway is stimulated using direct electric stimulation in neurosurgical patients.

At a theoretical level, it has been claimed that the temporary maintenance of serial order information could be performed without the need for specific item and serial order representational levels (Acheson & MacDonald, 2009; Jones & Macken, 2018; Schwering & MacDonald, 2020). A strong version of such an account considers that serial order information is exclusively maintained via the pattern of activations occurring within the linguistic system (Acheson, MacDonald, & Postle, 2011; Martin & Saffran, 1997; Poirier, Saint-Aubin, Mair, Tehan, & Tolan, 2015). For instance, according to Martin & Saffran (1997, p. 672):

“In principle, interactive activation processes could also play a role in maintaining serial order. The word node representing the first word in a sequence is primed first and therefore has more time to gain support from activated phonological and semantic representations compared to nodes that are primed later in a sequence. Thus, word nodes should show a gradient of activation levels across serial positions. […] Recency effects in supraspan recall reflect the increased phonological support that is due to the fact that at the time of recall, the activation levels of the terminal items have been less affected by the decay function inherent in the activation model.”

Likewise, Acheson et al. (2011, pp. 45–46) suggested that serial ordering errors could occur directly via an item’s relative level of activation in a language network:

“These interactive activation frameworks provide a potential explanation as to how semantic representation might influence the order of lexical-level utterance plans. When someone hears a word or a sequence of words, activation from that input simultaneously feeds forward to phonological representations and feeds back to semantic representations as well. After initial encoding, lexical activation is determined by repeated interaction with semantic and phonological representations. Serial ordering errors occur when the relative activation levels of the lexical items change because of this interaction.”

**Fig. 1:**
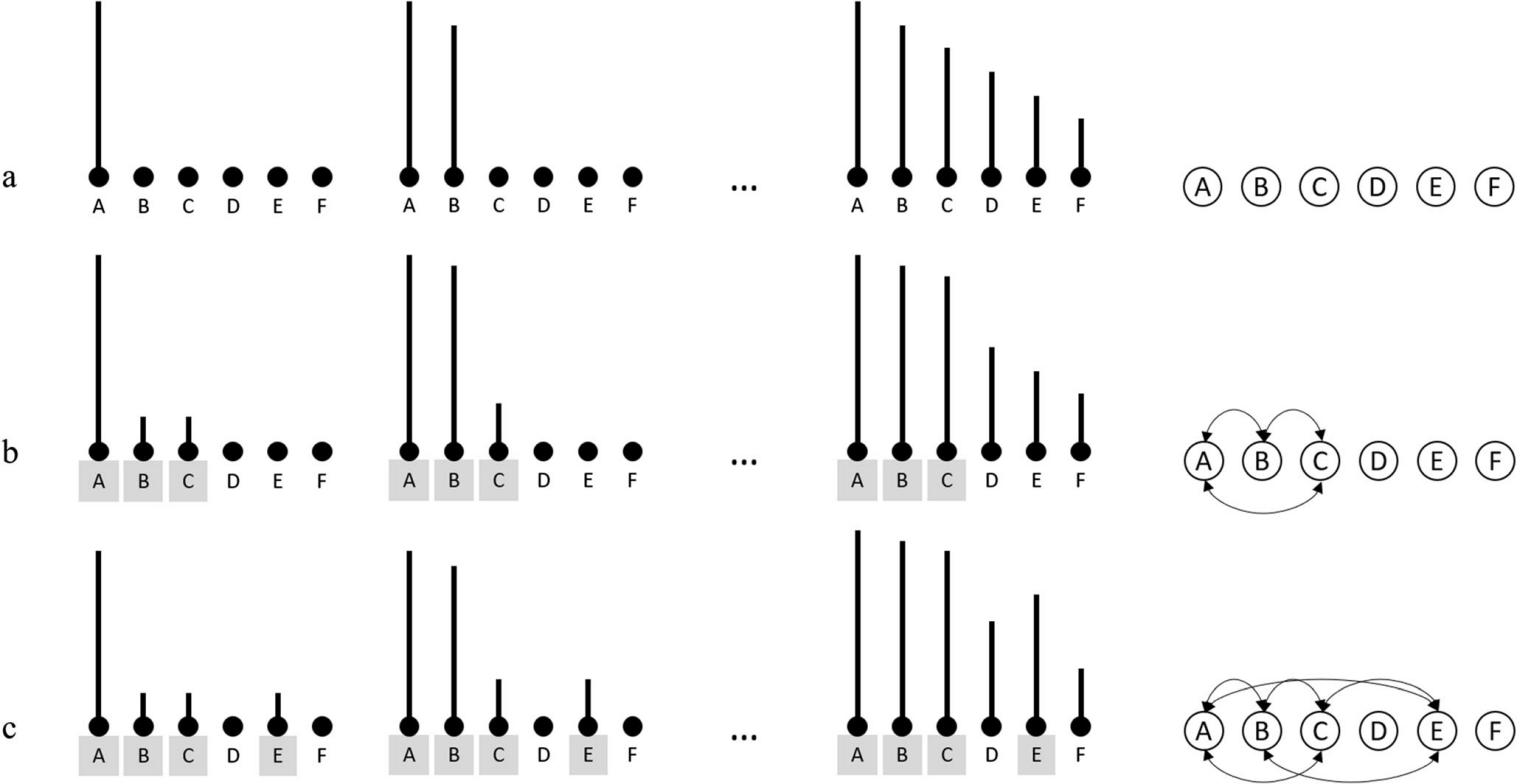
Illustration of the activation gradient (**a**) in a semantically unrelated condition, (**b**) in a condition in which items A, B, and C are semantically related, and (**c**) in a condition in which items A, B, C, and E are semantically related. Semantically related items are marked with an asterisk. As can be seen, the presence of semantic relatedness boosts the item's activation level for the related items

Based on this idea, Poirier et al. (2015) developed a more elaborate description of such models, termed the *ANet* account. According to this account, items in a to-be-remembered list are sequentially encoded in the linguistic long-term memory system with decreasing strength following an activation gradient,^1^ as displayed in Fig. 1. Serial order information is maintained via this activation gradient. Serial recall is performed by selecting the most strongly activated item at each recall attempt. Due to the selection mechanism being noisy, serial order errors eventually occur. An important prediction from this model is that modifying an item’s level of activation within the linguistic system should also affect the pattern of serial ordering errors in WM (Acheson et al., 2011).

Recent evidence appears to support this theoretical position. Poirier et al. (2015) manipulated semantic relatedness by presenting triplets of semantically related items in the first half of to-be-remembered lists. The subsequent items of the lists were semantically unrelated in the control condition (e.g., officer–badge–siren– music – tourist – yellow). In the experimental condition, the fifth item was semantically related to the triplet in the first half of the list. Compared to the control condition, the authors observed an increase of migration errors of the fifth item toward earlier serial positions, that is, towards the semantically related triplets of words. The authors assumed that since the semantically related triplets pre-activated, the semantically related target (i.e., via spreading of activation within a semantic network), this target should have a higher activation level in the experimental condition (Fig. 1c) as compared to the control condition (Fig. 1b). Since recall of serial order information is performed by selecting the most activated item, a gradient of activation in long-term memory could theoretically predict more migrations of the semantically related target toward earlier serial positions. As such, the manipulation of semantic relatedness is a critical and direct test of activation-based models, because it is supposed to modify the *relative* pattern of activation occurring within the linguistic system. This relative activation should in turn affect the processing of serial order information (Acheson et al., 2011), which the data of Poirier and colleagues appear to support. This was indeed a core prediction from their ANet account:

“In Experiment 1, we manipulated the level of activation of a target item to test the prediction that this would increase order errors for that item, making it likely that the CQ [Competitive Queuing] mechanism would select this item earlier because of its heightened activation; this early selection would mean that activation affected the order in which items were recalled.” (Poirier et al., 2015, p. 492).

Given that this theoretical account is in striking contrast with the majority of computational models of WM positing distinct item and serial order processing levels, the aim of the present study was to test the computational plausibility of a purely activation-based linguistic account for representing serial order information in a WM context. Most computational models of WM indeed explicitly assume the existence of serial order mechanisms that are distinct from those involved in item information. This is for instance the case as regards the TBRS* and SOB-CS architectures (Oberauer & Lewandowsky, 2011; Oberauer, Lewandowsky, Farrell, Jarrold, & Greaves, 2012), but also the computational models of Burgess and Hitch (1999, 2006) and Brown, Hulme, and Preece (2000). These types of architectures consider that serial order information is maintained via the creation of item-position associations, the positions being represented by specific representational mechanisms. These models, although strongly differing on the nature of the serial position representations, reliably reproduce important serial order phenomena, including primacy and recency effects and transposition error patterns.

In contrast, despite their conceptual simplicity and theoretical parsimony, purely activation-based linguistic WM architectures are rare and their ability for capturing serial order phenomena has not yet been assessed in a robust manner (see Norris, 2017, for a related discussion). In the present study, we implemented such an activation-based linguistic architecture, and we tested its ability to predict different effects involving the manipulation of semantic relatedness, which is a critical psycholinguistic factor to test the plausibility of a purely activation-based architecture. To overview the computational architecture, we first assumed serial order information as being maintained via a Primacy gradient of activation in long-term memory (Martin & Saffran, 1997; Page & Norris, 1998; Poirier et al., 2015). We then adapted this architecture by adding lateral excitatory connections to model semantic effects.

## Computational modeling

### Architecture

The architecture we used is a connectionist model composed of a single layer. When encoded, an item becomes active. This activation is supposed to occur directly in the long-term memory knowledge base. Semantically related items are connected via direct bidirectional excitatory connections, whose plausibility to model semantic effects in WM has already been demonstrated in three independent models (Haarmann & Usher, 2001; Kowialiewski & Majerus, 2020; Kowialiewski, Portrat, & Lemaire, 2021). The items are successively activated with decreasing strength using an activation gradient. Each encoded item automatically spreads activation towards the other semantically related items. Recall is performed by successively retrieving each item according to their activation value. For simplicity, we used the last implementation of the Primacy model, which was made available by Norris, Kalm, and Hall (2020). Our Julia implementation of the architecture we propose is freely available on the Open Science Framework (OSF): https://osf.io/9e4hu/.

#### Encoding

In the original Primacy model, encoding follows an activation gradient, which we denote *V*. This is defined by a *peak* value, γ, and a *step* value, α. The value γ is a free parameter and represents the starting value with which the first item is associated. The α value represents the amount of depletion from the γ value at each encoding stage. This parameter is fixed to 1. For instance, given a γ value of 20, the activation gradient is [20, 19, 18, 17, 16, 15] for a six-item list. Note that rehearsal is never explicitly modeled in the Primacy model. This includes the last implementation by Norris and colleagues. Activation within the model is simply derived from what would be expected if rehearsal theoretically occurs.

#### Spreading activation

During encoding, activation spreads toward semantically related nodes. This is modeled by including bidirectional excitatory connections. The strength of these connections is a free parameter, λ. At each encoding stage, items are activated using the activation gradient *V*. Activation then spreads bidirectionally within the network:

1 $$ {A}_{i,t}={A}_{i,t-1}+\sum \lambda .{A}_{j,t-1} $$

where *A*_*i*_ represents the final activation value associated to item i, and *A*_*j*_ is the activation coming from each semantically related item, j, scaled by the connection weight, *λ*. The subscript *t* represents the timestamp.

It is important to note that we do not intend to explicitly represent semantic knowledge. What we intend to represent through this spreading activation principle is the fact that semantically related items reactivate each other. In turn, this reactivation is supposed to modify item relative activation and therefore the pattern of serial order errors (Acheson et al., 2011). In other words, modifying the items’ relative level of activation in the semantic network also changes the model’s internal representation of their serial order.

#### Recall

After all items have been encoded, the model has to retrieve the items. This is made using a *competitive queuing mechanism.*^2^ Recall is a two-step process.

First, an item is selected as a potential candidate. This process is subject to noise:

2 $$ selectedItem={argmax}_i\left({A}_{i,t}+n\right)\  where\ n\sim N\left(0,\sigma \right) $$

This is modeled by adding temporary zero-centered random Gaussian noise to each item’s activation, with a standard deviation of *σ*, a free parameter. The most activated item is then selected. Response suppression (Duncan & Lewandowsky, 2005) already occurs at this stage, by setting the recalled item to a very low value (i.e., -999). This prevents the model from recalling an item twice.

Second, the activation value of the selected item is compared to an omission threshold. This threshold is drawn from a random Gaussian distribution *N*(*θ*, *σ*′), where *θ* and *σ*′ are two free parameters. If the selected item’s activation value (without the noise added during the first step) is above the retrieval threshold, the item is correctly recalled. Otherwise, an omission is produced. It must be pointed out that this implementation assumes response suppression as being always applied during the first step of retrieval, regardless of whether an omission had been produced during the second step. This choice of implementation by Norris et al. (2020) is unlikely to be plausible. But from the experience we gained by running the model many times, this is the only way the Primacy model can produce omission errors while modeling realistic serial position curves. Note that it is possible to produce realistic serial position curves while avoiding this implementation problem without affecting the model’s core assumptions. We, however, preferred to stick with the original implementation for simplicity.

By the time of each successive recall attempt, all items have decayed:

3 $$ {A}_{i,t}={A}_{i,t-1}.D $$

where *D* is a free parameter, ranging from 0 to 1. Due to this decay parameter, items recalled later in the lists are more subject to noise, because activation values converge towards an asymptote. All the parameters of the model are listed in Table 1.

**Table 1:** Annotations and values of the model

Symbol	Meaning	Value				
*α*	Step value depleted from γ	1				
*V*	Activation gradient	Depends on γ and *α*				
*A*	Items’ final activation	N/A				
Free parameters						
		Lower bound	Upper bound	Best parameters		
Symbol	Meaning			#1	#2	#3
*γ*	Peak activation value	10	30	14.444	12.761	14.444
*σ*	Noise added during selection	0	10	0.521	0.34	0.521
*θ*	Mean of the omission threshold	0	10	2.462	0.767	2.462
*σ’*	Noise of the omission threshold	0	10	7.518	9.569	7.518
*D*	Decay	0.1	0.9	0.853	0.71	0.853
*λ*	Lateral excitatory connections	0	0.1	0.01	0.0133	0.01

### Method

#### Datasets

The validity of this model was tested on three different sets of data: two datasets (Kowialiewski et al., 2021; Kowialiewski & Majerus, 2020) that include semantic and neutral conditions (i.e., the neutral condition being a semantically unrelated condition), and the data from Poirier et al. (2015), which we already described in the *Introduction*. The model is based on several parameters that partially depend on the task. Parameters were therefore estimated independently for each set of data. First, the parameters that do not depend on the semantic relatedness were estimated based on the neutral condition, in order to obtain a baseline model that would be able to reproduce standard serial-recall performance. Second, the semantic condition was used to estimate the λ parameter, which controls the level of semantic relatedness between items.

#### Overall scoring procedure

Serial position curves are plotted using a strict serial-recall criterion, in which an item is scored as correct only if it is recalled at the correct serial position. For instance, given the target sequence “Item1 – Item2 – Item3 – Item4 – Item5 – Item6” and the recall output “Item1 – Item2 – blank – Item3 – Item4 – Item6”, only Items 1, 2, and 6 would be scored as correct. To fit the experimental data, we also used an item-recall criterion, in which an item is scored as correct if correctly recalled, independently of its serial position. In the example mentioned above, items 1, 2, 3, 4, and 6 would be scored as correct. To assess the overall impact of semantic relatedness on order-recall performance, we computed an order-recall score for each experimental condition. This was done by dividing the number of time items have been recalled in the correct position (i.e., strict serial-recall criterion) by the number of times items have been recalled, regardless of their serial position (i.e., item-recall criterion).

#### Transposition rate

The pattern of transposition errors in the Poirier et al. (2015) study was plotted using transposition rates. We computed the number of transposition errors that occurred for item 5 (which is semantically related or not to items 1, 2, and 3), and for each position towards which item 5 could migrate. We then divided these numbers of transposition errors by the total number of times item 5 was recalled. This was computed separately for each experimental condition.

#### Parameter estimation

Estimation of the model’s basic parameters was performed using a simulated annealing algorithm (French & Kus, 2008; Kirkpatrick, Gelatt, & Vecchi, 1983) to find the lowest root mean square error (RMSE) between experimental and simulated serial position recall scores, on both strict and item-recall criteria. The RMSE was therefore always computed over 12 data points: six data points for the strict serial-recall criterion, and six data points for the item-recall criterion. The lower and upper boundaries of each free parameter are reported in Table 1. Estimation of the semantic parameter λ was much simpler and only required a grid search in [0,0.1] with a step of 0.0001. Importantly, λ was always estimated while keeping the model’s basic parameters constant. The value of λ that produced the smallest mean difference between the neutral condition and the experimental condition to the empirical data was then used. The idea was to select the value of λ that produces a difference between neutral and experimental scores similar to the human one. This was operationalized by minimizing the gap between the human mean difference and the model mean difference. We now present the three datasets as well as the simulations of these corresponding experiments. A summary of the different experimental conditions with study list examples is provided in Table 2.

**Table 2:** Examples of stimuli used across the three studies. Semantically related items are underlined

Study	Label	Item1	Item2	Item3	Item4	Item5	Item6
Kowialiewski and Majerus (2020)	Unrelated	Mouth	cousin	rain	dress	dog	bottle
	Related	piano	guitar	violin	flute	accordion	drums
Kowialiewski, Lemaire, & Portrat (2021)	NT	hammer	jacket	horn	wall	sky	dog
	T1	leaf	tree	branch	wall	sky	dog
	T2	wall	sky	dog	leaf	tree	branch
Poirier et al. (2015)	Control	officer	badge	siren	music	tourist	yellow
	Experimental	officer	badge	siren	fence	police	tractor

## Model assessment

### Dataset #1: Kowialiewski and Majerus (2020)

#### Data

This dataset was used to assess the model’s ability to reproduce the overall impact of semantic relatedness on serial-recall performance and order-recall performance. It is well established that semantic relatedness strongly enhances recall performance at the item level (see Kowialiewski & Majerus, 2020, for a meta-analysis). Semantic relatedness also has a small deleterious impact on the ability to recall serial order information, even though the effect is subtle (see also Ishiguro & Saito, 2020). Accordingly, we expect the architecture to have little or no impact on order-recall performance. We used the data reported in Kowialiewski and Majerus (2020), where they manipulated the semantic relatedness on six-item lists under interfering conditions or under immediate serial-recall tasks. Only the results from the latter condition were reported.

#### Model test

**Fig. 2:**
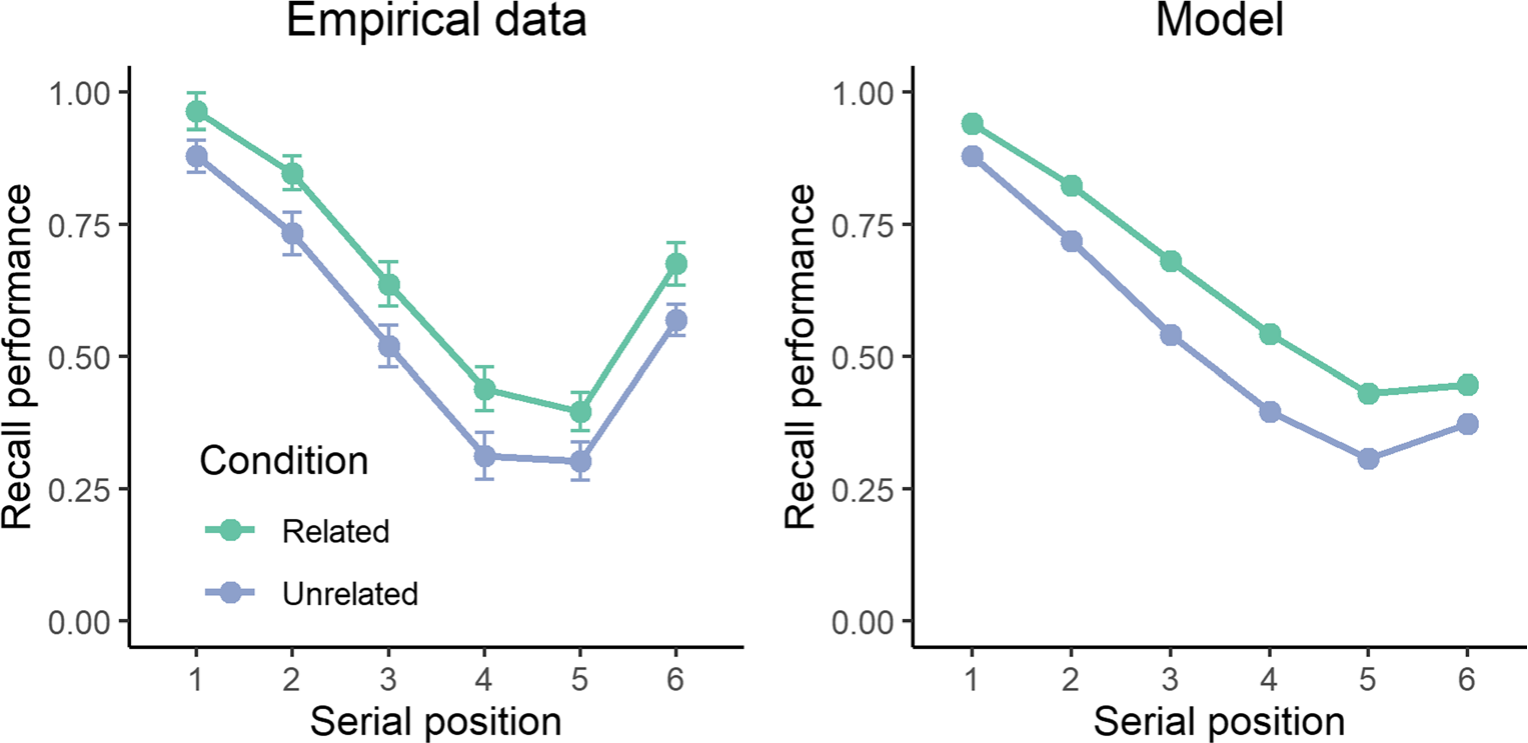
Recall performance across serial position for the empirical data (**left panel**) and the model (**right panel**) in the Kowialiewski and Majerus (2020) study. The experimental conditions involved the presentation of semantically related (green line) or unrelated (mauve line) items

**Fig. 3:**
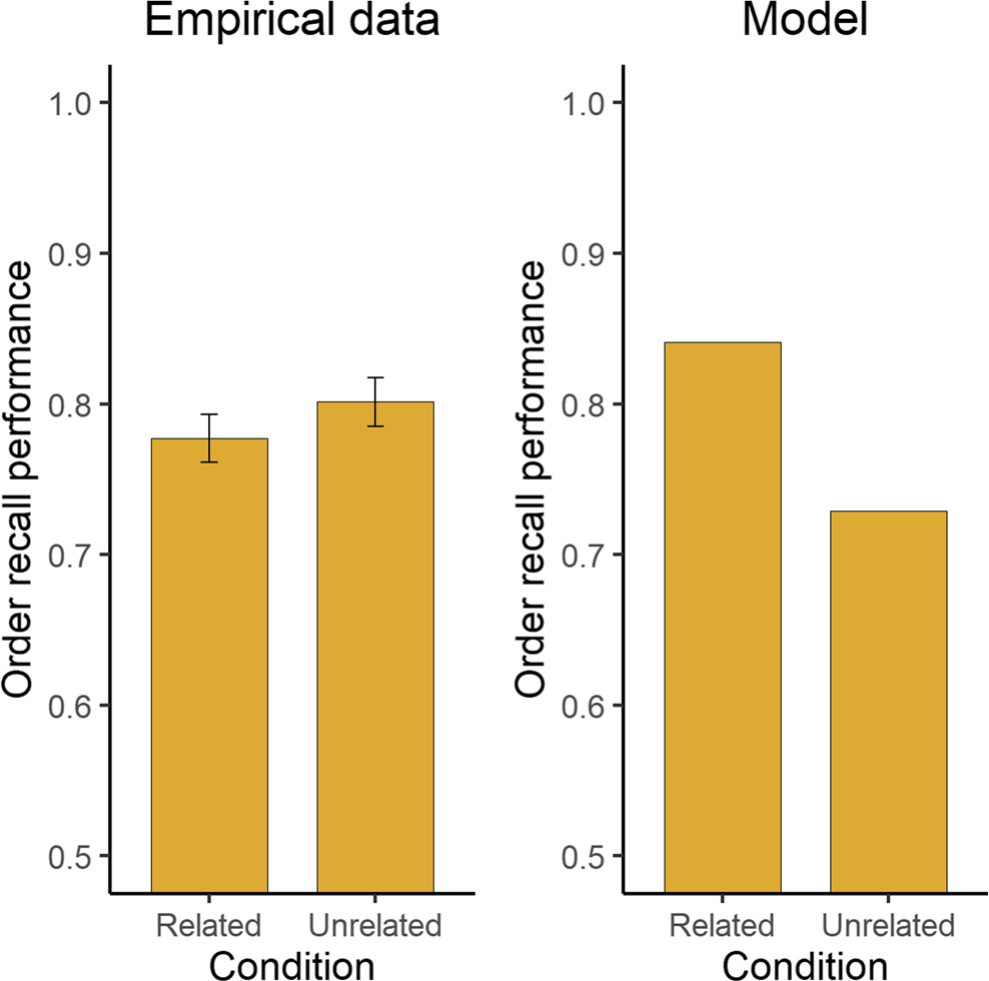
Order-recall performance as a function of semantic condition for the empirical data (**left panel**) and the model (**right panel**) in the Kowialiewski and Majerus (2020) study

In this study, the mean difference between the semantically related and unrelated conditions under a strict serial-recall criterion was 0.107. This value was used to estimate λ in the model. As can be seen in Fig. 2, the model reproduces the impact of the semantic relatedness dimension on overall recall performance. At the same time, semantic relatedness in the model also enhances order-recall performance (M = 0.841 and M = 0.729 in the related and unrelated conditions, respectively). This is not observed in the empirical data. Instead, order-recall performance among human subjects remains relatively unchanged (M = 0.782 and M = 0.812 in the related and unrelated conditions, respectively). The semantic effect found in the simulations (see Fig. 3) is a logical consequence of the model. As order-recall performance is driven by the activation level in long-term memory, increased activation leads to better between-item discriminability, and hence higher order-recall performance.

### Dataset #2: Kowialiewski, Lemaire, and Portrat (2021)

#### Data

It was recently shown (Kowialiewski et al., 2021) that the presence of semantic relatedness in a to-be-remembered list frees up WM resources, as previously observed using chunks (Portrat, Guida, Phénix, & Lemaire, 2016; Thalmann, Souza, & Oberauer, 2019). In this study, semantic relatedness was manipulated by presenting triplets of semantically related items either at the beginning (T1) or at the end (T2) of a six-itemto-be-remembered list. These conditions were compared to lists of unrelated items (NT). The semantically related triplets proactively enhanced recall performance for the subsequent, unrelated items, compared to the same items not preceded by a related triplet. However, the semantically related triplet did not retroactively impact recall performance. As we will see, the T2 condition is a critical test of the model.

#### Model test

**Fig. 4:**
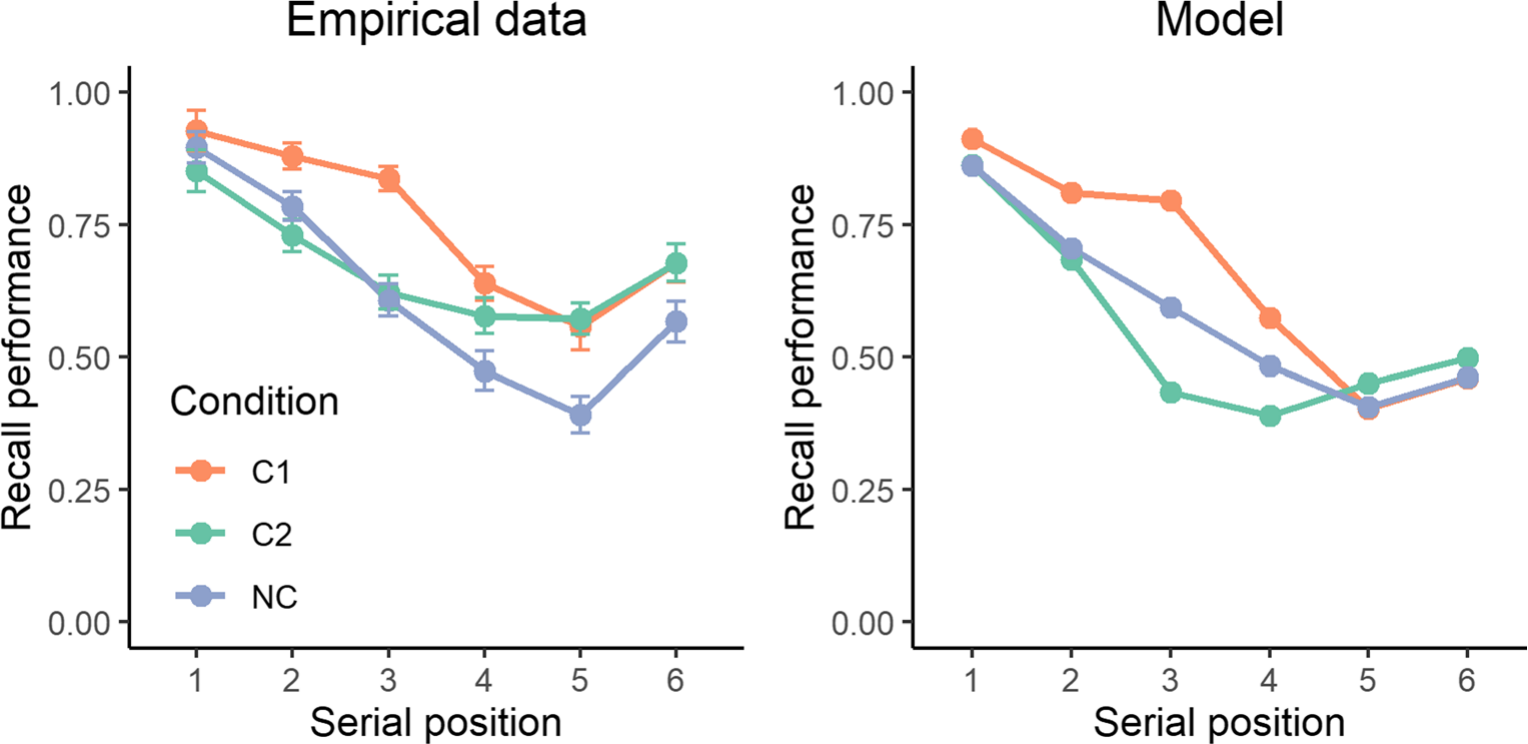
Recall performance across serial position for the empirical data (**left panel**) and the model (**right panel**) in the Kowialiewski, Lemaire, and Portrat (2021) study. The experimental manipulations involved the presence of semantically related triplets of words at the beginning (T1) or the end (T2) of the list, and compared this to a semantically unrelated condition (NT)

The mean difference between the T1 and NT conditions over positions 1, 2, and 3 was 0.122. This value was used to estimate λ in the model. Without modifying any of the basic parameters, the model predicts the recall advantage observed over the semantically related triplet in the T1 condition (Fig. 4). However, the model does not predict the proactive impact of semantic relatedness on the subsequent unrelated items. This latter result, we think, is not critical. Proactive effects could emerge by modeling maintenance mechanisms in a fine-grained manner (Portrat et al., 2016) or by including a limited-resource mechanism (Popov & Reder, 2020), which is beyond the scope of the present study. The critical result of these simulations is to show that the semantically related triplets have a retroactive deleterious impact on recall performance: when the semantically related triplet occurs at the end of the list (T2), recall performance of the third item is worse than in the neutral condition. This is a direct consequence of the modification of the pattern of activation in the model: since the related items in positions 4, 5, and 6 are more activated than other items, they are also more likely to be recalled towards earlier serial positions. In this case, items 3 and 4 are the most likely to be erroneously transposed due to their similar activation level. This pattern is absent in the empirical data. Instead, an absence of retroactive impact is observed.

### Dataset #3: Poirier et al. (2015)

#### Data

Poirier et al. (2015) observed that the manipulation of semantic relatedness changed the way items are transposed. In their Experiment 1, they presented items of which the first three were semantically related. The manipulation concerned the fifth item that was related as well in the experimental condition, as described in the *Introduction*. Critically, the fifth item was more often transposed towards position 3 in the experimental condition than in the control condition.

#### Model test

**Fig. 5:**
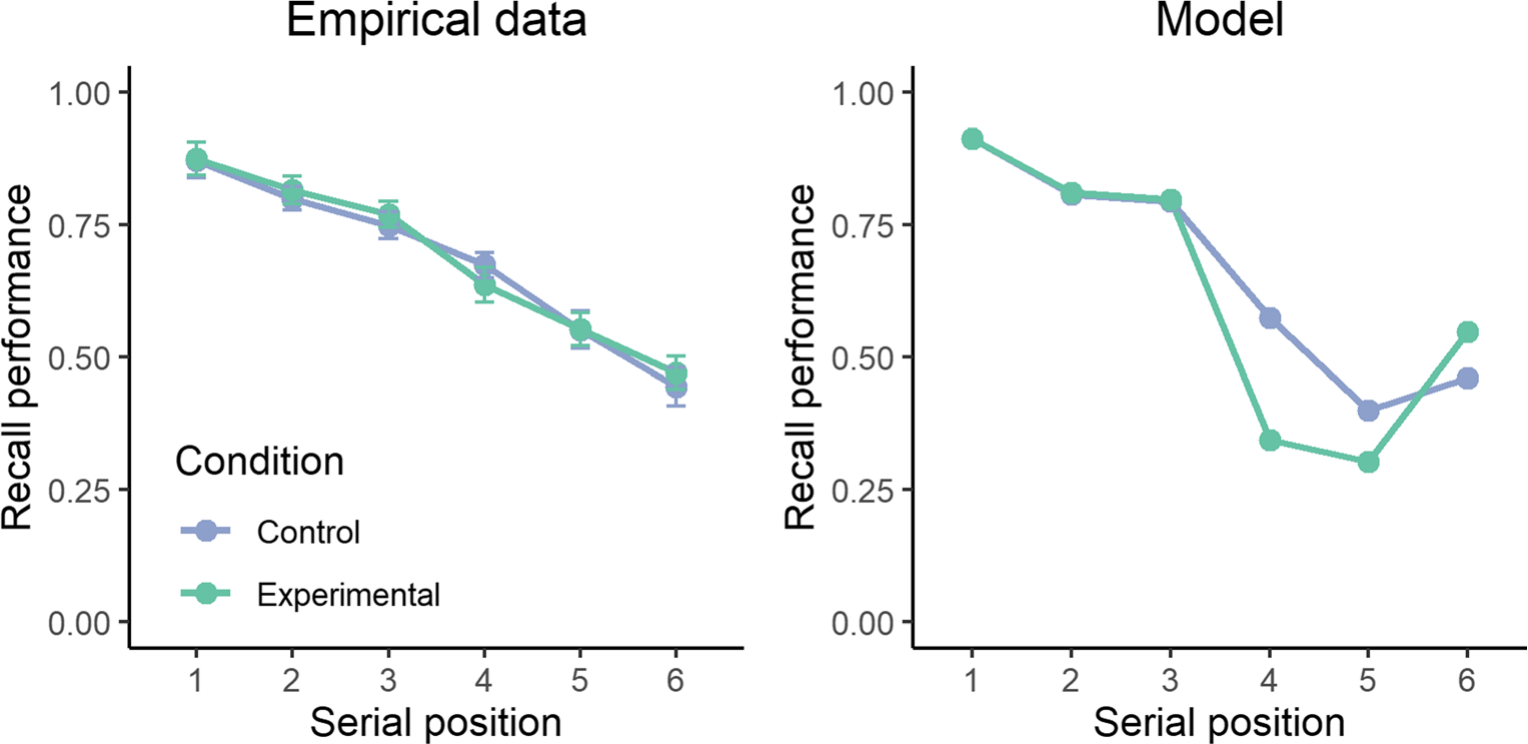
Recall performance across serial position for the empirical data (**left panel**) and the model (**right panel**). Each line represents the two experimental conditions originally manipulated by Poirier et al. (2015)

These data do not contain a non-semantic condition that prevents the estimation of the basic parameters of the model. We therefore reused the parameters estimated from the second dataset (see above). Figure 5 displays the results. The presence of semantic relatedness in the control condition (i.e., the first three items being semantically related) produces good patterns of recall performance. However, once the fifth item is semantically related to the triplet, a strong drop in performance is observed over positions 4 and 5.

**Fig. 6:**
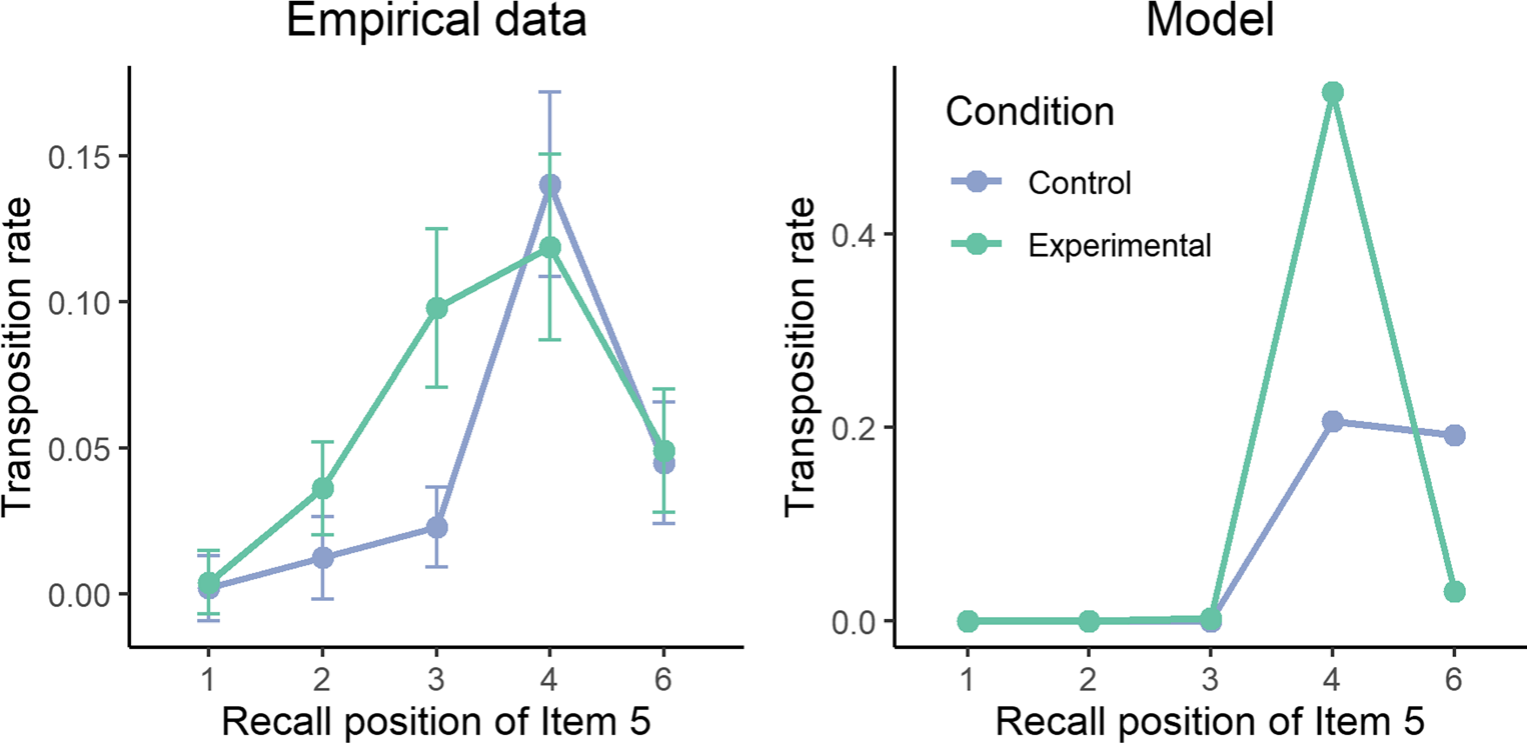
Transposition rate of item 5 across serial position for the empirical data (**left panel**) and the model (**right panel**)

This drop in performance over positions 4 and 5 in the experimental condition is explained by the pattern of serial order errors, displayed in Fig. 6. Critically, the vast majority of serial order errors in the experimental condition occurred in position 4. This is contrary to the empirical data where those transpositions tend to increase over position 3. This phenomenon is in fact a core property of the model: serial order errors are constrained by the pattern of activation in long-term memory. Increasing the activation level of one item results in an automatic and obligatory increase of transposition errors toward the directly preceding item.

One may argue that these results merely reflect the property of the model only for the specific set of parameters we found using the simulated annealing algorithm. Instead, there might be some configurations of parameters that could reproduce the results observed by Poirier et al. (2015). We explored this possibility by testing the model across a large sample of parameters. As reported in Appendix A, the model always fails to predict the increased transposition errors over position 3, and the absence of increased transposition errors over position 4.

## Discussion

This study used a computational modeling approach to investigate the hypothesis that serial order maintenance results from patterns of activation in long-term memory. The model successfully captured the overall impact of semantic relatedness effect on WM performance. However, it failed to predict the specific influence of semantic relatedness on the processing of serial order information. The model failed to predict the absence of semantic relatedness effects on order-recall performance, and instead predicted better order-recall performance for semantically related items. In addition, while human participants tend to erroneously recall an item at the position of its semantic neighbors, the computational model produced transposition errors involving migrations towards unrelated items.

Basically, the problem lies in the architecture's lack of dissociation between serial order information and the activation in long-term memory. Simulation of Dataset #1 showed that the increased level of activation of items led to better memory for order information, contrary to what is usually found in the literature (Ishiguro & Saito, 2020). This occurred in the model because the higher level of activation protects items against the deleterious effect of decay occurring at recall, decay following an exponential decay function as implemented in many computational architectures (Burgess & Hitch, 2006; Oberauer & Lewandowsky, 2011; Page & Norris, 1998). In addition, simulations of Dataset #2 and Dataset #3 suggest that if serial ordering errors are at least minimally constrained by an item’s relative level of activation in long-term memory, then a retroactive impact of semantically related items on recall performance should have been observed. Instead, empirical evidence from the WM literature converges towards an absence of retroactive impact of psycholinguistic factors on serial-recall performance (Cowan et al., 1992; Miller & Roodenrys, 2012; Portrat et al., 2016; Thalmann et al., 2019), a result that our activation-based architecture cannot reproduce. Hence, a purely activation-based architecture appears to be problematic to solve the problem of serial order in WM, contrary to what has been previously assumed (Acheson et al., 2011; Martin & Saffran, 1997; Poirier et al., 2015).

More generally, maintenance of serial order information via a primacy gradient in long-term memory is problematic for several reasons. First, it is not clear how the model would perform simple tasks, such as rehearsal/refreshing(Barrouillet, Bernardin, & Camos, 2004). This is because item selection is performed by choosing the most activated information. The model would be continuously stuck on the most activated item, which in most situations is the first one. Instead, participants can rehearse several items, and cumulatively (Tan & Ward, 2008). The original primacy model assumes rehearsal as being performed within a phonological loop (Page & Norris, 1998), but that does not solve the problem of serial order in the first place. Second, and as mentioned by Norris (2017), a purely activation-based model would never be able to recall an item twice (e.g., recalling “9-2-5-4-9-7”). This task requires temporary representations lying outside long-term memory. Note that this study does not rule out the Primacy model itself as a general mechanism through which serial order information could be coded outside long-term memory, as postulated by the original Page and Norris (1998) model. The present study simply rules out a primacy gradient of activation in long-term memory as an exclusive mechanism to maintain serial order information.

### How is serial order represented?

Many theoretical models of WM postulate an independence between the nature of the representational codes involved in maintenance of serial order information and those involved in item information. This relative independence is explicitly assumed by positional models of WM (Burgess & Hitch, 2006; Oberauer et al., 2012; Oberauer & Lewandowsky, 2011). Critically, these models should also consider potential interactions between item and serial ordering codes, but these interactions are not yet well understood. Jefferies et al. (2006) demonstrated a tendency for phonemes to migrate between nonwords, at the syllabic structure level (e.g., recalling “dug-fal” instead of “dag-ful”). Similarly, the pattern of transposition errors observed by Poirier et al. (2015) could be explained by assuming that WM also encodes semantic features. Due to the fact that semantically related items share overlapping semantic features and/or are also more similar (Dell, Schwartz, Martin, Saffran, & Gagnon, 1997; Ishiguro & Saito, 2020), transposition errors between semantically related items from more distant positions could theoretically occur at the moment of retrieval. Alternatively, as soon as participants detect the presence of a semantically related triplet, they might chunk the information and maintain a single semantic representation (e.g., “nature” instead of “leaf-tree-branch”). At recall, due to the decompression of the semantic chunk, the arbitrary order of the items themselves may be lost (Kowialiewski & Majerus, 2020), leading to the erroneous recall of a related word^3^.

This study fails to support the idea that serial order information is maintained via item-relative activation in the linguistic system. At the same time, this study does not discard the possibility that maintenance of serial order could be completely constrained by the statistical regularities learned from language exposure (Schwering & MacDonald, 2020). According to this account, the linguistic system possesses its own serial order maintenance mechanisms. This is, for instance, supported by studies showing that statistical regularities derived from linguistic corpora can predict serial-recall performance in verbal WM tasks (Jones & Macken, 2015, 2018). Critically, the plausibility of a purely language-based serial order maintenance mechanism has been demonstrated using a recurrent neural network (Botvinick & Plaut, 2006), the emerging behavior of which is shaped via the adjustment of connections weights using simple learning rules. At a conceptual level, this is radically different from the activation-based architecture we built, which is based on item-relative activation in long-term memory.

## Conclusion

This study looked at the range of plausible mechanisms involved in the temporary retention of serial order information. Through a computational modeling approach, we demonstrated that maintenance of serial order information via a primacy gradient of activation in long-term memory is implausible. Whether serial order is coded via independent serial order mechanisms or directly through the statistical regularities occurring in language processing or both remains to be formally established.

## Appendix A

In this analysis, we performed a grid search on 16,807 points of the parameter space of our model with *λ* = 0. The range of parameters explored is displayed in Table A1. We selected the combination of parameters (N = 13,103) that successfully reproduced a primacy effect on recall performance across both the strict serial-recall and item-recall criteria. The recency effect was not considered to ensure the analysis covered a broad range of points in the parameter space.

Next, we performed a new grid search on that combination of parameters by modulating *λ* (4 values) on each of them, both on the control and the experimental conditions from Experiment 1 of Poirier et al. (2015). Each set of parameters was estimated using 10,000 simulations. We then computed the number of transposition errors (corrected for the total number of times the target had been recalled) of the fifth item that occurred in positions 3 and 4 in each of these experimental conditions. For a result to be valid, we reasoned that there should be at least an increase of transposition errors in the experimental condition over position 3 superior to a potential increase of transposition over position 4. We counted the number of times these versions of the model corresponded to this criterion. There were none.

**Table 3:** Range of parameters explored in the grid search

Fixed parameters			
Parameter	Lower bound	Upper bound	Step
*γ*	10	22	2
*σ*	0.1	0.5	0.067
*θ*	1	5	0.667
*σ’*	4	8	0.667
*D*	0.55	0.95	0.067
*λ*	0.01	0.025	0.005

## Footnotes

1 This idea is directly derived from the Primacy model (Page & Norris, 1998). It is, however, important to note that the original Primacy model does not consider that this gradient directly occurs in long-term memory, contrary to the ANet account.

2 Poirier and colleagues suggested the competitive queueing mechanism as being modeled using an accumulator model, following Hurlstone and Hitch (2015). We implemented such a competitive queueing mechanism based on accumulator principles (available on OSF). This did not provide any enhancement of the model whatsoever, with the exception that the accumulator model provides the further opportunity to make predictions on recall latencies, which is beyond the purpose of this study. Therefore, we simply stuck with the last available implementation of the Primacy model.

3 A positional explanation can also propose that a semantically related triplet is linked with one given positional tag, which creates the possibility of order errors within the triplet, whereas semantically non-related elements have their own tag, which minimize order errors.
